# DNA Methylation Variation Is Identified in Monozygotic Twins Discordant for Non-syndromic Cleft Lip and Palate

**DOI:** 10.3389/fcell.2021.656865

**Published:** 2021-05-12

**Authors:** Juan I. Young, Susan Slifer, Jacqueline T. Hecht, Susan H. Blanton

**Affiliations:** ^1^John P. Hussman Institute for Human Genomics, Dr. John T. Macdonald Foundation Department of Human Genetics, Miller School of Medicine, University of Miami, Miami, FL, United States; ^2^McGovern Medical School, University of Texas Health Science Center, Houston, TX, United States

**Keywords:** methylation, NSCLP, non-syndromic cleft lip and cleft palate, twins, whole genome bisulfite sequencing

## Abstract

Non-syndromic cleft lip with or without cleft palate (NSCLP) is the most common craniofacial birth defect. The etiology of NSCLP is complex with multiple genes and environmental factors playing causal roles. Although studies have identified numerous genetic markers associated with NSCLP, the role of epigenetic variation remains relatively unexplored. Because of their identical DNA sequences, monozygotic (MZ) twins discordant for NSCLP are an ideal model for examining the potential contribution of DNA methylation to non-syndromic orofacial clefting. In this study, we compared the patterns of whole genome DNA methylation in six MZ twin pairs discordant for NSCLP. Differentially methylated positions (DMPs) and regions (DMRs) were identified in NSCLP candidate genes, including differential methylation in *MAFB* and *ZEB2* in two independent MZ twin pairs. In addition to DNA methylation differences in NSCLP candidate genes, we found common differential methylation in genes belonging to the Hippo signaling pathway, implicating this mechanosensory pathway in the etiology of NSCLP. The results of this novel approach using MZ twins discordant for NSCLP suggests that differential methylation is one mechanism contributing to NSCLP, meriting future studies on the role of DNA methylation in familial and sporadic NSCLP.

## Introduction

Non-syndromic cleft lip with and without cleft palate (NSCLP) is a common birth defect occurring in 1/700-1000 livebirths, affecting 135,000 newborns worldwide. The severity varies based on laterality (bi- versus unilateral involvement) and extensiveness (involves only the lip, the primary palate and/or the secondary palate). The defect affects feeding initially and, in the long term, speech articulation, hearing, dental hygiene, dentition and overall well-being as there is always residual scarring. The cost of multiple surgical interventions exact a large emotional burden for patients and families, and high financial burden for families and society.

Despite many decades of research, only limited environmental agents and genetic causes of NSCLP are known (Chiquet et al., 2011; Letra et al., 2012, 2014; Cvjetkovic et al., 2015; Leslie et al., 2015, 2016a,b; Fish et al., 2017; Ludwig et al., 2017; Cox et al., 2018). While candidate gene and genome-wide studies of case-control and family-based datasets have identified causal/susceptibility loci and variants, the majority of the genetic causes remain elusive. Based on the analysis of multiplex families, in which there are multiple, but not necessarily first degree, affected relatives, there are likely extra-genic factors affecting penetrance and/or expression of the genetic liability. For example, MZ twins have a concordance rate of » 25–50% for NSCLP (compared to 1–3% in DZ twins) with a heritability estimate of approximately 70%, indicating that other factors play a role (Christensen, 1999; Grosen et al., 2011). It is likely that variation in multiple genes is etiologically necessary, with extra-genic factors contributing with a threshold effect.

Epigenetics is well-recognized as a critical mediator of the interplay between genes and the environment and influences phenotypic outcomes. DNA methylation, a major substrate of epigenetic information, regulates genome activity and provides an alternative mechanism for modulating cellular physiology compared to those arising solely from genetic changes (Esteller, 2008). Thus, a potential mechanism by which environmental factors could elicit altered tissue morphogenesis is variation in gene methylation. Support for this hypothesis comes from the observation that maternal use of the methyl-group donor folic acid may decrease the risk for NSCLP (Butali et al., 2013; Wallenstein et al., 2013).

Methylation differences within normal MZ twin pairs are well documented (Kaminsky et al., 2009; Czyz et al., 2012; Ollikainen et al., 2015). Importantly, methylation differences have been identified in MZ twin pairs discordant for a variety of congenital defects, including Beckwith-Wiedemann syndrome (OMIM 130650), congenital cataracts, congenital renal agenesis, cryptorchidism, Mayer-Rokitansky-Kuster-Hauser syndrome (OMIM 277000) and congenital heart disease (OMIM 217095), yielding insights into the underlying pathology (Weksberg et al., 2002; Rall et al., 2011; Tierling et al., 2011; Jin et al., 2014; Wei et al., 2015; Lu et al., 2017; Lyu et al., 2018). Given the relevance of DNA methylation in neural crest development, which plays a critical role in craniofacial development (Hu et al., 2014; Seelan et al., 2019), and the potential influence of epigenetics in the etiology of NSCLP, we compared genomic DNA methylation patterns between six pairs of monozygotic twins discordant for NSCLP.

## Materials and Methods

### Editorial Policies and Ethical Considerations

All DNA samples were collected and studied following informed and signed written consent from a parent or guardian. This study was approved by the University of Texas Health Science Center Committee for Protection of Human Subjects (HSC-MS-03-090) and the University of Miami Human Subject Research Office (IRB #20100505).

### Dataset

Six monozygotic twin pairs discordant for NSCLP were ascertained and characterized as part of the larger genetic study of NSCLP that has been previously described (Chiquet et al., 2007; Morris et al., 2020). Briefly, participants were recruited at the craniofacial clinics affiliated with the UTHealth Science Center at Houston under IRB-approved protocols and written informed consent was obtained from parents and assents from the children. All participants were evaluated and syndromic forms of NSCLP were excluded. Study inclusion was irrespective of ethnicity and/or sex. Family history and a minimum of a three-generation pedigree were obtained from all families. The characteristics of the six pairs of MZ twins and their samples are listed in Table 1. All affected individuals were post-surgical repair. Saliva samples were collected using the Oragene^®^ Saliva Collection Kit or the Oragene^®^ Saliva Self−Collection Kit for Young Children (Sponge Kit) according to manufacturer’s protocol. Genomic DNA was isolated from saliva samples obtained from these male MZ twins discordant for NSCLP and from their available parents.

**TABLE 1:** Description of twin pairs.

Pair*	**Sex**^#^	**Status**^&^	**Age (yrs)**^∧^	**Cleft type**	**Ethnicity**^@^
MZ1	M	A	8	Left	NHW
	M	U	8	–	
MZ2	M	A	1	Left	H
	M	U	1	–	
MZ3	M	A	10	Left	H
	M	U	10	–	
MZ6	M	A	8	Left	H
	M	U	7	–	
MZ7	M	A	2	Left	NHW
	M	U	2	–	
MZ8	M	A	15	Left	NHW
	M	U	15	–	

*All samples were saliva. MZ, monozygotic; **^#^**M, male; ^&^A, affected; U, unaffected;**^∧^**Age, Age at sampling; **^@^**NHW, Non-Hispanic White; H, Hispanic.

### Candidate Gene Assembly

A list of candidate genes for NSCLP was assembled from a thorough review of the literature of orofacial clefting including human association and sequencing studies and animal models. Genes associated with non-syndromic cleft lip with or without cleft palate, cleft palate only, and syndromic forms of clefting were included (Supplementary Table 1).

### Whole Genome Sample Preparation and Sequencing

gDNA samples were evaluated for concentration by Qubit^®^ fluorometric assay or by using Picogreen^®^. The integrity of the gDNA was assessed by loading approximately 100 ng per sample on a 0.75% agarose gel and comparing size distribution to a size marker (Quick-Load^®^ 1 kb Extend DNA Ladder, NEB). 0.6–1 μg of gDNA was fragmented using the Covaris L-series or E-series instrument and sheared to a final insert size of approximately 400 bp. The purified fragmented DNA was repaired by the phosphorylation of the 5′ end and the conversion of overhangs to blunt ends. One “A” nucleotide was added to each 3′ end of the blunt fragments. The indexing-specific paired-end adaptors were ligated to the ends of the DNA fragments and the adaptor-ligated library was PCR amplified. Quality and quantity of the purified ligated products were verified using the Caliper LabChip^®^ GX. Libraries were pooled and sequenced according to manufacturer’s instructions *via* the Illumina HiSeq X platform. Multiplexing 6 whole human genomes per flow cell generated an average depth of 30× per sample, exceeding read depth recommendations for Whole genome bisulfite sequencing (WGBS) analysis (Ziller et al., 2015).

Comparison of the whole genome sequencing (WGS, Table 2) between the affected and unaffected co-twins did not identify a pathogenic point mutation, insertion/deletion, or structural alteration in any of the known CLP-associated genes listed in Supplementary Table 1.

**TABLE 2:** Whole genome DNA sequencing statistics.

Average reads uniquely aligned to human genome*	**Average genome coverage**	**Average discordant SNP calls**	**Average discordant exonic/splicing SNP calls**	**Discordant SNP calls in NSCLP candidate genes**
9.19E + 10 (8.61E + 10–1.01E + 11)	29.12 (28.42–32.52)	196,253 (187,112–213,468)	14,920 (13,435–19,423)	0 (0–0)

*Range in brackets.

### Whole Genome Bisulfite Sequencing

Whole genome bisulfite sequencing gDNA library preparations were carried out using the TruSeq DNA Sample Prep Kit v2 (Illumina Inc.) combined with sodium bisulfite conversion. Briefly, 1 μg of gDNA spiked with 5 ng unmethylated λ DNA (Promega) was fragmented to 200–300 bp peak size (Covaris, Woburn, MA, United States). Fragment size was analyzed on a Bioanalyzer DNA 1000 Chip (Agilent). End repair, sample purification with AMPure beads (Agencourt Bioscience Corp.), adenylation of 3′ ends, and adaptor ligation were carried out following the Illumina standard protocol. Ligation DNA was purified with AMPure, analyzed on a Bioanalyzer and subjected to bisulfite conversion using the MethylCode Bisulfite Conversion Kit (ThermoFisher) according to manufacturer’s instructions. Adaptor-ligated DNA was amplified through five cycles of PCR using the Hifi Uracil + DNA polymerase (Kapa Biosystems, Woburn, MA, United States) according to manufacturer’s protocol. Library quality was monitored using the Bioanalyzer (Agilent) and the concentration of fragments carrying adapters at both ends determined by quantitative PCR using the Library Quantification Kit from KAPA Biosystem. Paired-end DNA sequencing (124 bp each) using the Illumina Hi-Seq 2500 was performed.

Generated sequencing reads (average read length 120.9) were trimmed using Trim Galore^1^ and aligned to the bisulfite-converted human reference genome (GRCh37/hg19) using Burrows–Wheeler alignment. Methylation states were called using Bismark v0.10.0 (Krueger and Andrews, 2011). Confirmed clonal reads and reads with low mapping quality score were removed (Johnson et al., 2012), as well as, reads aligning to regions of the reference genome identified as troublesome for high- throughput sequencing aligners [DAC Blacklisted Regions (DBR) and Duke Excluded Regions (DER)^2^].

After data preprocessing, »54.3% of the resulting reads were uniquely mapped to the human reference genome. The median bisulfite conversion efficiency of CpGs was determined to be ∼98%. CpGs not covered by at least 20 reads were excluded, which resulted in a minimum of two reads per strand across all twelve samples. Reads on opposite strands with an absolute strand difference in methylation > 20% were excluded, leaving on average 2.4 × 10^6^ unique and high-confidence CpGs per sample. Most CpG sites retained were highly methylated (over 50%, Supplementary Figure 1).

### Array-Based Methylation Assay

Array-based methylation profiling was performed using the MethylationEPIC BeadChip (Illumina, San Diego, CA, United States) assay. Briefly, 300 ng gDNA per sample were bisulfite converted using the EZ-96 DNA Methylation kit (Zymo Research, Irvine, CA, United States) and processed as described by Illumina. The MethylationEPIC BeadChip measures over 850,000 CpG loci across a diverse set of functionally relevant genomic regions that include CpG islands (CGI) and promoters as well as CGI shores, gene body and intergenic CpGs. BeadChip images were scanned and the data analyzed using the R software (version 3.2.4^3^). Raw intensity files (.idat) were obtained and processed using RnBeads package (Assenov et al., 2014). For quality control, we selected probes that had a detection p-value < 0.01 for all samples. The detection p-value represents the quality of the probes compared to background noise. Because all samples were male, we did not filter probes associated with sex. Methylated and unmethylated signal intensities were quantile normalized for each probe type separately and both beta and M values were estimated. The beta values are the ratio of the methylated signal intensity to the sum of both methylated and unmethylated signals after background subtraction (beta values range from 0 to 1, corresponding to completely unmethylated and fully methylated sites, respectively). M values are logit transformation of the beta values, which is *M* = log (beta/1-beta). M values were used as outcome variables for subsequent analysis because they have been shown to have better statistical properties such as homoscedasticity (Du et al., 2017). For the analysis of individual CpGs, an absolute delta beta > 0.30 was used to detect differential methylation between twins.

### Identification of Differentially Methylated Positions Within Twin Pairs

From the strand-overlapping sites with > 20 reads, we selected differentially methylated sites based on the following criteria: (1) intra-twin pair difference in methylation levels of at least 30% (delta methylation > | 0.3|) and (2) a coefficient of variation in methylation levels of the unaffected individuals of less than 0.25. This strategy of restricting the search on differential methylation to sites in which the variation in unaffected twins is <25% is based on the idea that sites that exhibit large inter-individual variation in methylation levels have less regulatory potential than those sites in which the methylation levels are more tightly preserved between unaffected individuals.

### DMR Analysis

Differentially methylated regions (DMR) analysis used a Bayesian hierarchical model (Wu et al., 2015) to detect regional methylation differences with ≥ 3 contiguous sites each with a methylation difference > 20% within a 2 kb distance with unidirectional methylation changes and a region-wise average methylation difference > 25%.

### DNA Bisulfite Conversion and Bisulfite Specific PCR

Flanking primers (methylation-unbiased nested PCR primers) were designed for a subset of differentially methylated CpG sites and quantitative levels of methylation for each CpG dinucleotide were evaluated using the BiQ Analyzer software. Bisulfite-PCR was followed by cloning and Sanger sequencing of multiple clones for short-range mapping of methylation levels in selected loci. Genomic DNA (0.25 μg) was bisulfite converted using the EpiTect Kit (Qiagen) following the manufacturer’s protocol. Converted DNA was amplified with primers designed in MethPrimer. Optimized PCR reactions were followed by cloning using the TOPO TA Kit (Invitrogen). Multiple clones were sequenced. Bisulfite-cloning validated 70% of the DMS (Supplementary Figure 2).

### Pyrosequencing Validation

Locus-specific pyrosequencing was conducted to validate the methylation data at selected loci. Pyrosequencing assays were designed and implemented by EpigenDx (Worcester, MA, United States) following the manufacturer’s recommended protocol. The correlation between the WGBS and pyrosequencing data was evaluated using the Spearman rank order correlation test. Pyrosequencing validated 73% of the DMS. Interestingly, the majority of validated DMSs had contiguous CpGs that also showed differential methylation in the same direction and with a similar magnitude to the targeted CpG.

## Results

### DNA Methylation Profiles Are Different in Monozygotic Twins Discordant for NSCLP

**FIGURE 1:**
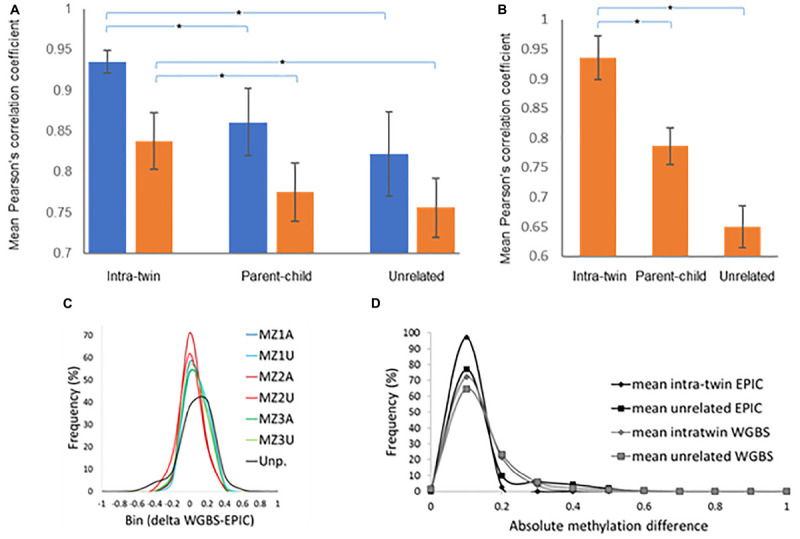
MZ co-twins are highly correlated for DNA methylation but have site-specific differences. **(A)** Pearson’s correlation coefficients derived from comparing intra-twin, parent-child and parent to parent (unrelated) methylation levels at 100X (blue) or 30X (brown) genome coverage. **(B)** Correlation coefficients using only variable CpGs as identified by Feinberg et al. (2010) at 30X genome coverage. **(C)** Comparison between DNA methylation values for 16,920 individual CpG loci generated by MethylationEPIC microarray and whole-genome bisulfite sequencing. Methylation values generated by Rnbeads for the MethylationEPIC microarray were subtracted from methylation levels calculated from the sequencing data for the same sample (MZ1A to MZ3U). Averaged comparisons contrasting EPIC and WGBS data from unpaired (Unp.) samples is also included (black line). **(D)** Distribution of average absolute differences in DNA methylation within the MZ twins as well as comparisons of unrelated individuals. A significantly (P<1.2e-10 for WGBS and P<1.9E-12 for EPIC, Kolmogorov–Smirnov test) smaller number of CpG sites with a large average within-twin differences in methylation level was observed in within twin comparisons as contrasted with comparisons of unrelated individuals (unaffected-unaffected, NSCLP-unaffected (inter-twins) or NSCLP-NSCLP).

Methylome analysis was performed on saliva samples from six sets of male MZ twins discordant for NSCLP using whole-genome bisulfite sequencing (WGBS). Globally, DNA methylation profiles generated by WGBS were highly correlated across all individuals, indicating inter-individual conservation in methylation levels. The correlation of methylation patterns of the co-twins was significantly higher compared to unrelated individuals, indicative of low within-pair DNA methylation variation (average *r* = 0.84 for twins and *r* = 0.75 for unrelated individuals at 30X coverage; Figure 1A and Supplementary Figure 3). Removal of areas of extreme variability across individuals (variably methylated regions, VMRs) (Feinberg et al., 2010) from the analysis of the 30X coverage increased the correlation for intra-twin comparisons (average *r* = 0.93), but decreased it for unrelated samples (average *r* = 0.65) (Figure 1B). This pattern of correlation change suggests that the polymorphic nature of these VMRs is affected to a larger extent by environmental rather than genetic influences.

The WGBS data was validated globally by subjecting DNA from three of the MZ pairs to the MethylationEPIC microarray (Illumina) assay. This assay interrogates the DNA methylation status of ∼865,000 CpG sites, which account for only ∼3% of all human autosomal CpGs. The methylation values obtained from the microarray were correlated with those from WGBS. WGBS data (prefiltered as described in the Methods section) were further filtered to include only CpGs present in the MethylationEPIC microarray, resulting in a total of 16,920 loci for comparison (Figure 1C). The mean methylation level correlation between WGBS and the microarray determinations for the six samples was *r* = 0.90 and, on average, 97.63% of loci have delta methylation levels < | 0.2|, indicating that the methylation values derived from the sequencing and the array methods are consistent across commonly interrogated loci.

To identify CpGs displaying differential methylation in any of the discordant twin pairs, the differences in CpG methylation between any two paired samples were estimated. Figure 1D shows the distribution of the mean absolute differences in DNA methylation levels per individual CpG within twins, as well as between unrelated samples. The overall distribution of average intra-twin DNA methylation differences showed a significant skew to the left as compared to the distribution of the average unrelated pair differences, with fewer CpG sites having large average differences in DNA methylation (Figure 1D). However, there is still variability in DNA methylation levels at individual CpG sites within the pairs of MZ twins.

### DNA Methylation Differences in MZ Twin Pairs

The differentially methylated CpG sites (DMSs) identified within twin pairs is presented in Supplementary Table 2. The number of DMS per MZ pair varies from 7 (MZ2) to 42(MZ1), for a total of 151 DMSs associated with 147 genes. No single CpG is differentially methylated in more than one twin pair. This lack of overlap in DMS per pair is also seen at the gene level; there are no shared genes containing DMSs in the different MZ pairs. Table 3 lists the five genes with the most differential methylation per twin pair.

**TABLE 3:** Five most differentially methylated CpGs for each NSCLP twin pair.

	**Chr**	**Position**	**Gene 1**	**Gene 2**
MZ1	16	84479557	*TLDC1*	*ATP2C2*
	10	24754430	*KIAA1217*	*ARHGAP21*
	10	24754448	*KIAA1217*	*ARHGAP21*
	6	33518020	*BAK1*	*ZBTB9*
	16	23296275	*SCNN1B*	*SCNN1G*
MZ2	12	4141700	*CCND2*	*PARP11*
	15	48849784	*FBN1*	*DUT*
	8	28206128	*PNOC*	*ZNF395*
	12	4141713	*CCND2*	*PARP11*
	6	129494272	*LAMA2*	*ARHGAP18*
MZ3	X	85175162	*POF1B*	*CHM*
	1	234913189	*IRF2BP2*	*TOMM20*
	15	41104572	*DNAJC17*	
	15	41104527	*DNAJC17*	
	1	45280071	*TCTEX1D4*	*PTCH2*
MZ6	2	132153021	*TUBA3D*	*POTEE*
	5	10869280	*DAP*	
	7	50850334	*GRB10*	*COBL*
	7	50850302	*GRB10*	*COBL*
	22	30617445	*LIF*	*HORMAD2*
MZ7	Y	15676869	*TMSB4Y*	*UTY*
	Y	15676854	*TMSB4Y*	*UTY*
	6	28663545	*SCAND3*	*TRIM27*
	1	52616167	*ZFYVE9*	*CC2D1B*
	1	52616219	*ZFYVE9*	*CC2D1B*
MZ8	8	71368648	*NCOA2*	*TRAM1*
	16	2141571	*NTHL1*	*PKD1*
	5	1331076	*TERT*	*CLPTM1L*
	7	150822700	*AGAP3*	*GBX1*
	7	150822698	*AGAP3*	*GBX1*

To evaluate epigenetic involvement of candidate NSCLP genes, we tested whether any of the identified DMSs in the discordant twins were located in known or suspected NSCLP genes (Supplementary Table 1). Evidence of aberrant methylation in single CpGs was observed in seven NSCLP genes, four in MZ1, two in MZ2 and one in MZ6 (Table 4). Note that the number of DMSs in candidate genes is more than expected by chance in MZ1 (*p*-value < 0.003) and in MZ2 (*p*-value < 0.0001).

**TABLE 4:** Differentially methylated positions in candidate genes identified in three NSCLP twin pairs.

**MZ pair**	**Gene**	**Chr**	**Position**	**Delta methylation**	Genomic feature*	**Distance to TSS (kb)^#^**	**Candidate group^&^**
MZ1	*TP63*	3	189509045	0.80	In (alt. promoter)	1.6	AGV
	*WNT7A*	3	13787881	−0.78	IGR	133	AGV
	*MAFB*	20	39373248	−0.84	IGR	−55	AGV
	*GDF6*	8	96365815	0.85	IGR	807	AGV
MZ2	*MAF*	16	79211133	0.63	IGR	400	AGV
	*FBN1*	15	48849784	−0.78	In	88	SCLP
MZ6	*SOX9*	17	71010900	−0.59	IGR	890	SCLP

*In, Intron; IGR, intergenic region; **^#^**TSS, transcriptional start site; Genome assembly, GRCh37/hg19; **^&^**AGV, associated genetic variant; SCLP, syndromic cleft lip and palate.

DNA methylation levels are strongly correlated among neighboring sites across the genome, and identification of clusters of differentially methylated positions rather than single CpGs usually predicts functional relevance (Eckhardt et al., 2006). Regional analysis is also less prone to be affected by the technical artifacts associated with individual sites. Thus, we performed systematic regional analyses of the WGBS data to identify differentially methylated regions (DMRs) in the MZ NSCLP discordant twins. Amongst the six MZ pairs, we identified a total of 768 DMRs, varying in numbers from 61 in MZ2 to 349 in MZ1 (Supplementary Table 2). Table 5 lists the 10 most differentially methylated DMRs per MZ twin pair. Candidate genes containing DMRs are listed in Table 6. While each twin pair had DMRs in one or more candidate genes, only twin pairs MZ1 MZ2, and MZ3 had an overrepresentation of candidate genes among all their respective DMRs (MZ1 *p* < 0.004, MZ2 *p* < 0.02, and MZ3 *p* < 0.0006). A gene-based overlap analysis detected only two candidate genes containing DMRs in more than a single MZ pair, *MAFB* (MZ6 and MZ8) and *ZEB2* (MZ3 and MZ8). The DMRs in *ZEB2* are in two different areas of intron 2 (AB011141) while the DMRs in *MAFB* are overlapping and located in the proximal upstream region.

**TABLE 5:** Top ten differentially methylated regions in each NSCLP twin pair.

	**Chr**	**Start**	**End**	**# CpGs**	**Gene 1**	**Gene2**
MZ1	10	133992228	133994340	25	*DPYSL4*	*JAKMIP3*
	1	231475643	231476069	20	*EXOC8*	*SPRTN*
	2	206723874	206723953	6	*NRP2*	*INO80D*
	22	30309896	30310188	9	*HORMAD2*	*MTMR3*
	2	95537267	95537391	11	*TEKT4*	
	17	46632154	46632211	7	*HOXB3*	
	4	185251292	185251345	7	*ENPP6*	*IRF2*
	9	136405836	136405917	6	*ADAMTSL2*	*FAM163B*
	10	69664114	69664588	7	*SIRT1*	*HERC4*
	17	35285044	35285218	9	*LHX1*	*MRM1*
MZ2	12	4141672	4141751	5	*CCND2*	*PARP11*
	19	38803473	38804151	5	*YIF1B*	*C19orf33*
	7	2832917	2832990	10	*GNA12*	*AMZ1*
	17	14358178	14358230	4	*HS3ST3B1*	*PMP22*
	17	57860285	57860352	7	*VMP1*	*TUBD1*
	2	223481094	223481197	7	*FARSB*	*SGPP2*
	6	158619635	158619734	6	*TULP4*	*GTF2H5*
	21	19274639	19274704	7	*CHODL*	*C21orf91*
	12	64216292	64216411	6	*SRGAP1*	*TMEM5*
	2	131852087	131852175	6	*FAM168B*	
MZ3	15	41104483	41104658	7	*DNAJC17*	
	1	45280056	45280131	8	*TCTEX1D4*	*PTCH2*
	1	11178496	11178611	8	*ANGPTL7*	*EXOSC10*
	22	19825520	19825636	6	*C22orf29*	*TBX1*
	11	44359724	44359809	6	*CD82*	*ALX4*
	16	85033868	85033924	4	*ZDHHC7*	*CRISPLD2*
	7	137154759	137154861	5	*PTN*	*DGKI*
	12	69069932	69070032	5	*NUP107*	*RAP1B*
	17	48520295	48520396	6	*ACSF2*	*CHAD*
	17	77638489	77639656	6	*RBFOX3*	*ENPP7*
MZ6	6	169977358	169977440	12	*THBS2*	*WDR27*
	19	2960100	2960479	11	*TLE6*	*ZNF77*
	19	13129284	13129382	6	*NFIX*	*LYL1*
	6	28945493	28945566	10	*TRIM27*	*ZNF311*
	16	15083564	15083666	9	*PDXDC1*	*NTAN1*
	8	54442087	54442157	8	*OPRK1*	*ATP6V1H*
	22	30617390	30617470	7	*LIF*	*HORMAD2*
	14	71721206	71721284	9	*SIPA1L1*	*PCNX*
	12	3948535	3948614	9	*EFCAB4B*	*PARP11*
	6	24647358	24647446	8	*KIAA0319*	
MZ7	6	28663014	28665327	29	*SCAND3*	*TRIM27*
	Y	15676834	15676905	7	*TMSB4Y*	*UTY*
	6	25882373	25882434	8	*SLC17A3*	*SLC17A2*
	3	169377635	169378355	11	*MECOM*	*ACTRT3*
	1	154908464	154908782	8	*PMVK*	
	5	15008177	15008861	7	*FBXL7*	*ANKH*
	17	56634655	56634821	8	*SEPT4*	*TEX14*
	X	2994672	2994725	7	*ARSF*	*MXRA5*
	9	138339180	138339399	8	*PPP1R26*	*OLFM1*
	8	141220160	141220218	6	*TRAPPC9*	*C8orf17*
MZ8	10	135343183	135343281	16	*CYP2E1*	*SYCE1*
	11	71210236	71210315	9	*KRTAP5-7*	*NADSYN1*
	9	139546440	139546629	11	*NOTCH1*	*EGFL7*
	22	46279171	46279335	9	*WNT7B*	*ATXN10*
	5	171541730	171541852	11	*FBXW11*	*STK10*
	3	107416865	107416968	9	*BBX*	*CD47*
	2	68907054	68907187	7	*ARHGAP25*	*PROKR1*
	7	5205136	5205268	7	*WIPI2*	*RBAK*
	22	23605028	23605090	9	*BCR*	*IGLL1*
	1	64135554	64135639	6	*ROR1*	*PGM1*

**TABLE 6:** Differentially methylated regions identified in candidate genes.

**Chr**	**Start**	**End# CpG**	**Delta methylation**	**Gene**	**distance to TSS**	Genomic feature*	**Candidate group^&^**
MZ11	2976318	297910310	0.50	*PRDM16*	−8	IGR	MGI
1	55359014	553591535	−0.51	*DHCR24*	−6	IGR	SCLP
1	93425751	934258035	0.50	*RPL5*	128	In	SCLP
1	243451450	2434515444	−0.53	*SDCCAG8*	32	In	MGI
2	114099657	1141003026	−0.59	*PAX8*	−63	IGR	AGV
3	13787881	137879714	−0.78	*WNT7A*	134	IGR	AGV
3	41282685	412832684	−0.52	*CTNNB1*	42	IGR	AGV
3	129328813	1293291188	0.54	*PLXND1*	−3	IGR	MGI
3	189509045	1895095354	0.58	*TP63*	160	In	AGV
5	149861203	1498614154	−0.51	*NDST1*	−26	IGR	MGI
8	96365815	963658694	0.85	*GDF6*	807	IGR	SCLP
10	131555549	1315558496	−0.51	*MGMT*	290	In	AGV
12	99652840	996528965	0.54	*APAF1*	614	In	MGI
12	125008438	1250117835	0.55	*NCOR2*	30	In	SCLP
13	106929698	1069302854	0.55	*EFNB2*	257	IGR	MGI
14	37123643	371237005	−0.62	*PAX9*	−7	IGR	MGI
14	53976736	539769235	0.64	*BMP4*	447	IGR	AGV
15	26877653	268778594	0.54	*GABRB3*	140	In	MGI
16	79361536	793616375	0.60	*MAF*	273	IGR	AGV
17	42646731	426467965	−0.66	*FZD2*	12	IGR	MGI
17	72038593	720406385	0.53	*RPL38*	−160	IGR	MGI
18	46345407	463455535	0.52	*SMAD7*	132	In	MGI
22	34207931	342113294	−0.50	*LARGE*	107	In	AGV
MZ21	87668656	876687255	−0.63	*HS2ST1*	288	IGR	MGI
1	164904923	1649049784	−0.54	*PBX1*	376	IGR	AGV
3	57741006	577431715	0.50	*SLMAP*	0	Prom	MGI
4	157863234	1578633284	−0.55	*PDGFC*	29	In	AGV
6	44424513	444245704	−0.54	*RUNX2*	−872	IGR	MGI
14	29568885	295695974	−0.56	*FOXG1*	334	IGR	SCLP
MZ32	145216430	1452167095	−0.58	*ZEB2*	62	In	MGI
5	176551269	1765513574	−0.52	*NSD1*	−10	IGR	SCLP
9	17005412	170055224	0.58	*BNC2*	−135	IGR	MGI
16	4000600	40006524	0.54	*CREBBP/ADCY9*	−70/166	IGR	AGV
16	85033868	850339244	0.73	*CRISPLD2*	180	In	AGV
17	44918054	449198024	−0.53	*WNT3/WNT9B*	−23/−10	IGR	AGV
22	19825520	198256366	−0.62	*TBX1*	81	In	AGV
22	46438139	464382034	0.51	*WNT7B*	−65	IGR	AGV
MZ61	156095505	1560956085	0.63	*LMNA*	11	In	MGI
2	43859116	438592215	0.52	*THADA*	−36	IGR	AGV
9	129250270	1292503326	−0.57	*LMX1B*	−126	IGR	SCLP
11	61143440	611435494	0.56	*TMEM216*	−16	IGR	SCLP
15	28338841	283388985	0.50	*OCA2*	6	In	MGI
20	39319752	393209396	−0.68	*MAFB*	−2	Prom	AGV
MZ713	22473353	224734475	0.51	*FGF9*	228	IGR	MGI
2	158607860	1586080196	0.55	*ACVR1*	124	In	MGI
21	43048389	430485686	0.50	*RIPK4*	139	IGR	SCLP
MZ820	39320842	393210114	−0.59	*MAFB*	−3	IGR	AGV
17	8869579	88696508	−0.51	*PIK3R5*	−1	Prom	AGV
16	68829893	688299584	0.51	*CDH1*	59	In	AGV
2	145247233	1452472934	0.60	*ZEB2*	31	In	MGI

TSS, transcriptional start site; Genome assembly, GRCh37/hg19; *In, Intron; IGR, intergenic region; Prom, promoter; **^&^**AGV, associated genetic variant; SCLP, syndromic cleft lip and palate; MGI, Mouse Genome Informatics (orofacial cleft phenotype).

### Enrichment of Identified DMRs in Shared Disease-Relevant Pathways

To identify enriched molecular pathways, EnrichR (Chen et al., 2013; Kuleshov et al., 2016) was used to analyze the DMR-gene set from each twin pair (Table 7). Across the six twin pairs, comparison of the top 10 significantly enriched Kegg pathways from all differentially methylated genes identified common enrichment of the “Hippo Signaling Pathway” in three pairs (MZ1, MZ3 and MZ8) and “Signaling pathways regulating pluripotency of stem cells” in two pairs (MZ1 and MZ3). Interestingly, pathway analysis using only our candidate genes identified enrichment in Hippo signaling in MZ1 (BMP4, FZD2, WNT7A, CTNNB1, GDF6, and SMAD7) and Wnt signaling in MZ3 (CREBBP, WNT9B, and WNT3). Additionally, we found that the combined set of genes identified as differentially methylated in any twin pair (set of 1,234 genes) was significantly enriched in the terms Osteoblast differentiation (*p* < 6.6E-8), and Neural crest migration (*p* < 4.1E-4) (Supplementary Figure 4).

**TABLE 7:** Pathways enriched for differentially methylated genes*.

**Index**	**Name**	**Adjusted *p*-value**
**MZ1**		
1	Signaling pathways regulating pluripotency of stem cells	0.009879
2	Thyroid hormone signaling pathway	0.01003
3	Longevity regulating pathway	0.0102
4	Transcriptional misregulation in cancer	0.01558
5	Small cell lung cancer	0.01709
6	Hippo signaling pathway	0.02574
7	Longevity regulating pathway	0.0261
**MZ3**		
1	Wnt signaling pathway	0.0008854
2	Melanogenesis	0.001263
3	Pathways in cancer	0.001309
4	Insulin secretion	0.00152
5	Glucagon signaling pathway	0.002417
6	Pancreatic secretion	0.002557
7	Cushing syndrome	0.002559
8	Basal cell carcinoma	0.002761
9	Salivary secretion	0.01002
10	cAMP signaling pathway	0.01172
11	Aldosterone synthesis and secretion	0.01216
12	Gastric cancer	0.01276
13	Hippo signaling pathway	0.01431
14	Gastric acid secretion	0.02412
15	Thyroid hormone synthesis	0.02429
16	Oocyte meiosis	0.02514
17	Relaxin signaling pathway	0.02519
18	Vascular smooth muscle contraction	0.02559
19	Cell cycle	0.02588
20	p53 signaling pathway	0.02664
21	Estrogen signaling pathway	0.02739
22	Signaling pathways regulating pluripotency of stem cells	0.02791
23	Apelin signaling pathway	0.0287
24	Colorectal cancer	0.02993
25	Adrenergic signaling in cardiomyocytes	0.03086
**MZ8**		
1	Hippo signaling pathway	0.03994

*No significant enrichment in any Kegg pathways was identified in MZ2, MZ6, and MZ7.

### Between-Group Analyses Identified Additional DMRs

Group level DNA methylation differences between NSCLP cases and controls were also examined. Unlike the within-pair discordant MZ twin design, between-group DNA methylation differences can be attributed to both genetic and epigenetic factors. This analysis identified significantly different methylation regions only in the genes KCNAB3, NWD1, IFLTD1, and TRBJ2-3. No DMRs in NSCLP candidate genes were identified in this analysis.

## Discussion

This is the first comprehensive study of epigenetic differences in MZ twins discordant for NSCLP. This epigenome-wide study was performed using saliva DNA from six male MZ twin pairs discordant for NSCLP to determine if differences in DNA methylation could explain the discordant NSCLP phenotype. Results from this study provide novel and exciting findings which point to the importance of methylation in craniofacial development.

We found regional methylation differences in a number of genes in each twin pair (range 61*–*349). Less than 3% (37 out of 1,338) of these genes were differentially methylated in more than one twin pair. A single gene, TRIM27, was differentially methylated in three pairs. TRIM27 has not been previously associated with clefting or craniofacial development but has been shown to be differentially methylated in prostate and lung cancers (Ji et al., 2020; Wang et al., 2020). Interestingly, differential methylation of TRIM27 was reported in a study comparing cleft lip only to cleft palate only (Sharp et al., 2017).

When we restricted our analysis to NSCLP candidate genes, evidence of methylation differences was found, suggesting their possible contribution to NSCLP through epigenetic variation. Of the 49 candidate genes which exhibited differential methylation, only two, MAFB and ZEB2, were differentially methylated in more than one twin pair. Importantly, the changes were all in the same direction, suggesting that these two genes are epigenetically important in orofacial development. This provides additional support for the previously reported association between NSCLP and MAFB, which is expressed in palatal shelves (Beaty et al., 2010). ZEB2 is involved in embryonic development of facial features (Ghoumid et al., 2013; Funato and Nakamura, 2017), and Zeb2 mutant mice exhibit alterations in neural crest migration and midfacial clefting (Van de Putte et al., 2003; Miyoshi et al., 2006). The lack of significant gene overlap points to the underlying heterogeneity of NSCLP.

Interestingly, although the majority of the identified DMRs do not lie in genes previously associated with NSCLP, pathway enrichment analysis revealed overrepresentation of the Hippo pathway in three of the six twin pairs. This signaling pathway was initially discovered as a key regulator of tissue growth in flies and regulates organ size and tissue repair by controlling cell proliferation, survival, mobility, stemness, and differentiation. The Hippo pathway is tightly regulated by both intrinsic and extrinsic signals, such as mechanical force, cell–cell contact, polarity, energy status, stress, and many diffusible hormonal factors. Studies have shown evidence of crosstalk between the Hippo pathway and other key signaling pathways, such as Wnt signaling (Li et al., 2019), and has recently been associated with craniofacial development (Wang et al., 2016; Wang and Martin, 2017; Sun et al., 2018) and mouse secondary palate formation (Goodwin et al., 2020). The common enrichment in this pathway is not due to alterations in a small subset of similar genes in all pairs (see Supplementary Figure 5). Rather, there is pathway convergence through alterations in MZ pair-specific methylation in different genes. A comparison of our results with a recent orofacial clefting case-control report revealed a little overlap (Alvizi et al., 2017). Interestingly, this overlap includes the NSCLP candidate gene MAFB (Supplementary Table 3). The different findings between these studies provides further evidence for complex genetic and epigenetics involvement in the etiology of NSCLP.

It is well known that genetic variation in a single gene can cause syndromic cases of orofacial clefting. *It is therefore possible that extreme epigenetic variation in a few key genes could produce a clefting phenotype. However, it is more likely that the effects of most DNA methylation changes are subtle, similar to some non-coding genetic variants, and that multiple changes act in concert to ultimately contribute to non-sydromic orofacial clefting, as discussed above*. The observation of DMPs in genes previously implicated in NSCLP, such as TP63, TBX1, CRISPLD2, CREBBP, and BMP4, suggest that *de novo* epimutations, which could imitate genetic mutations (Martin et al., 2005), are likely involved in the pathogenesis of NSCLP. *Since we found methylation changes in several NSCLP candidate genes in each affected twin, we propose that an “epigenetic load” mechanism is involved in the dysregulation of orofacial morphogenesis, rather than a single dysfunctional NSCLP gene. This is consistent with the accepted multifactorial inheritance of NSCLP* (Carter, 1969; Hecht et al., 1991; Howe et al., 2018). Based on the important role of epigenetic mechanisms in regulating gene expression, it is likely that methylomic variation could mediate disease susceptibility by cumulative altered gene expression, similar to genetic variation in regulatory regions of the genome.

The use of saliva as a proxy for the lip and palatal tissues is justified by their common developmental origin (Patel and Hoffman, 2014). *Salivary glands, lip, and palate all arise from neural crest cells, making cells from saliva a developmentally appropriate proxy for these tissues.* In addition, saliva is a good source of high quality DNA for use in epigenomics (Thompson et al., 2013; Smith et al., 2015) and has been deemed more informative than blood for DNA methylation analysis (Lowe et al., 2013) in spite of the possibility of bacterial contamination resulting in non-human reads in human sequencing data (Samson et al., 2020).

Our sample size, while small, exceeds the number of twins in most epigenetic studies of birth defects with most being based on a single discordant twin pair (Weksberg et al., 2002; Rall et al., 2011; Tierling et al., 2011; Jin et al., 2014; Wei et al., 2015; Lu et al., 2017; Lyu et al., 2018). NSCLP has an estimated frequency of 1 in 700; thus, discordant MZ twin pairs occur infrequently but are rich source of information.

The stringent parameters used in this study, together with the validation of selected DMPs by either bisulfite sequencing, pyrosequencing, or methylation arrays, suggest that these are likely real differences rather than reflecting experimental noise. Amongst the identified methylation differences between co-twins, we propose that many of them underlie the discordance in NSCLP but recognize that there will be some that may reflect individual exposure to environmental factors unrelated to NSCLP, or perhaps non-craniofacial phenotypic differences. However, identification of DMRs in NSCLP candidate genes and the pathway analyses of the unbiased genes containing DMRs suggest that these DMRs play an etiologic role in NSCLP.

In summary, we show for the first time that MZ twins discordant for NSCLP have differential methylation. The findings from this novel study demonstrate that methylation may play a significant role in the etiology of NSCLP and, more importantly, differential methylation could account for the “reduced penetrance” seen in many multiplex families (Hecht et al., 1991; Hecht and Blanton, 1998). *Since the* discordant twins share age, sex and socioeconomic status and do not exhibit CLP-related genotype variability (as analyzed by WGS), our data highlights the role of non-shared environmental and stochastic factors in the etiology of epigenetic differences and resulting discordant NSCLP phenotypes. Importantly, we find methylation differences in genes previously implicated by NSCLP genetic studies. Moreover, our data suggest that although DNA methylation at some CpG sites is consistently altered in affected twins, most differences are individual-specific, revealing epigenetic heterogeneity between NSCLP individuals. This suggests that each affected individual/family may have their own unique epigenetic signature making etiologic identification complex. However, the differences converge at the level of potentially affected pathways including the Hippo signaling pathway. Our findings complement a recent NSCLP case-control study suggesting that epigenetic mechanisms underlie NSCLP (Alvizi et al., 2017). Future studies are aimed at defining epigenetic changes in sporadic cases, as well as in multiplex NSCLP families in which DNA methylation patterns can be traced through multiple generations.

## Data Availability Statement

The data presented in the study are deposited in the Gene Expression Omnibus (GEO) repository, accession number GSE173211.

## Ethics Statement

The studies involving human participants were reviewed and approved by this study was approved by the University of Texas Health Science Center Committee for Protection of Human Subjects (HSC-MS-03-090) and the University of Miami Human Subject Research Office (IRB #20100505). Written informed consent to participate in this study was provided by the participants’ legal guardian/next of kin.

## Author Contributions

JY, JH, and SB: conceptualization, investigation, writing—original draft, and writing—review and editing. JY and SS: formal analysis and methodology. All authors contributed to the article and approved the submitted version.

## Conflict of Interest

The authors declare that the research was conducted in the absence of any commercial or financial relationships that could be construed as a potential conflict of interest.

## Footnotes

1 https://www.bioinformatics.babraham.ac.uk/projects/trim_galore/

2 http://hgwdev.cse.ucsc.edu/cgi-bin/hgFileUi?db=hg19&g=wgEncodeMapability

3 https://www.r-project.org/
