# Supplementary material for: DNA Methylation Variation Is Identified in Monozygotic Twins Discordant for Non-syndromic Cleft Lip and Palate
Source: Front Cell Dev Biol. 2021 May 12;9:656865. doi: 10.3389/fcell.2021.656865 (PMC8149607; doi:10.3389/fcell.2021.656865)
Supplement: Supplementary file 1 [file Data_Sheet_1.docx]

**Supplemental Figure S1. Differential distribution of DNA methylation levels in WGBS and EPIC array**. Histograms show bimodal distribution pattern of DNA methylation profiles in affected and unaffected individuals analyzed by both, WGBS and array. Depiction of the frequency of CpGs according to DNA methylation levels identifies the differences in sites probed by the two methods. Although the vast majority of CpGs in the human genome are methylated, the biased inclusion of CpG islands in the array-based method is reflected in a strongly bimodal distribution (right panels). Conversely, WGBS has amplification bias of methylated DNA compared to unmethylated sequences, leading to overrepresentation of methylated sites (left panels). Twin pairs are represented by colors. Top panels show a binned distribution of the data in bar graph and bottom panels in continuous lines.


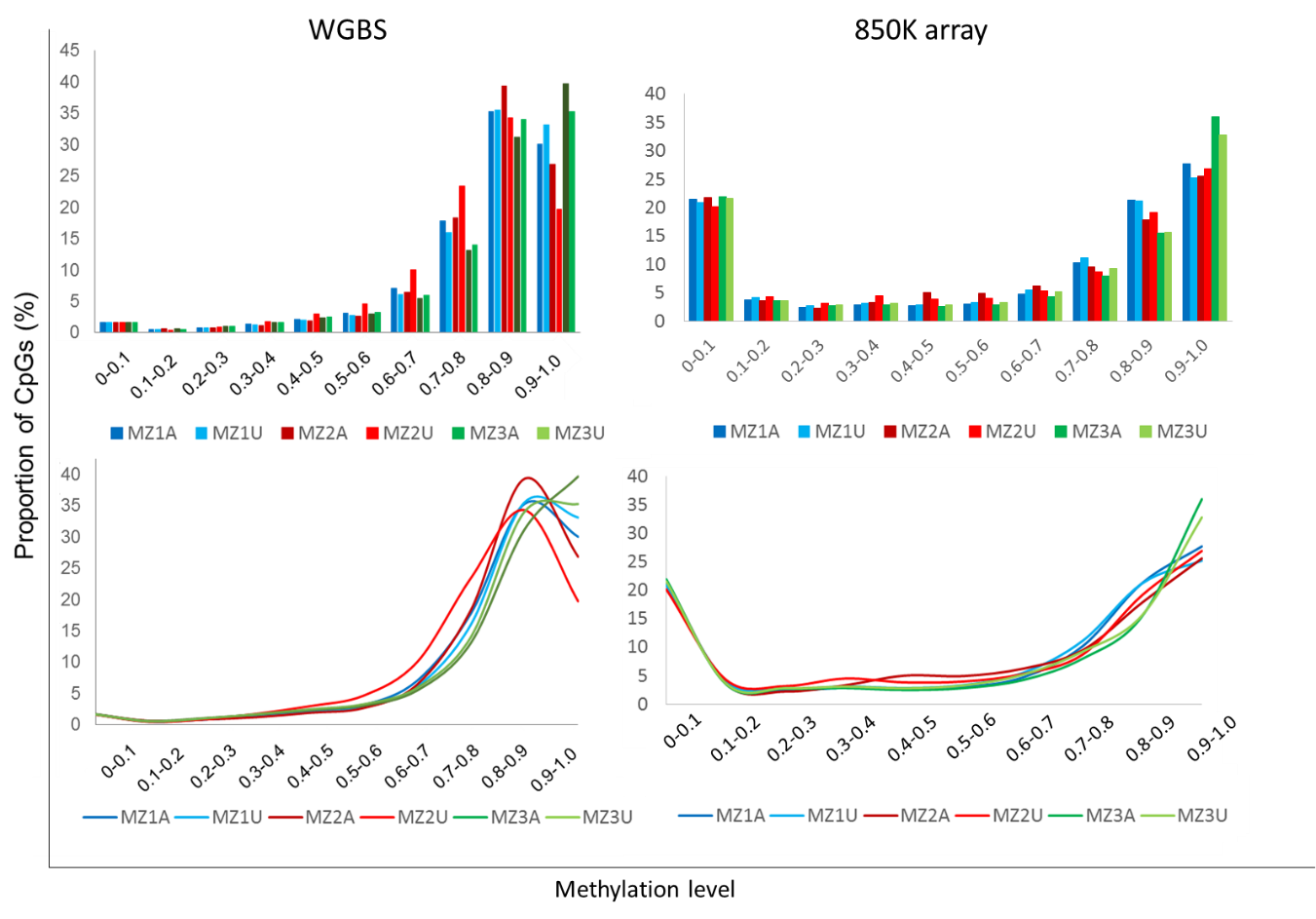


**Supplemental Figure S2. Validation of WGBS data using site specific sequencing demonstrated concordant differential methylation in selected CpGs**. Validation of DNA methylation differences was performed using a site-specific sodium bisulfite sequencing method (orange markers) and pyrosequencing (blue markers). An average of 12 alleles were Sanger sequenced for each group and the average difference among groups plotted in the X axis. The Y axis represent the methylation differences in the corresponding MZ group as derived from the WGBS data. Diamond, circle and square dots correspond to validation for MZ1, MZ2 and MZ3 pairs, respectively.

**
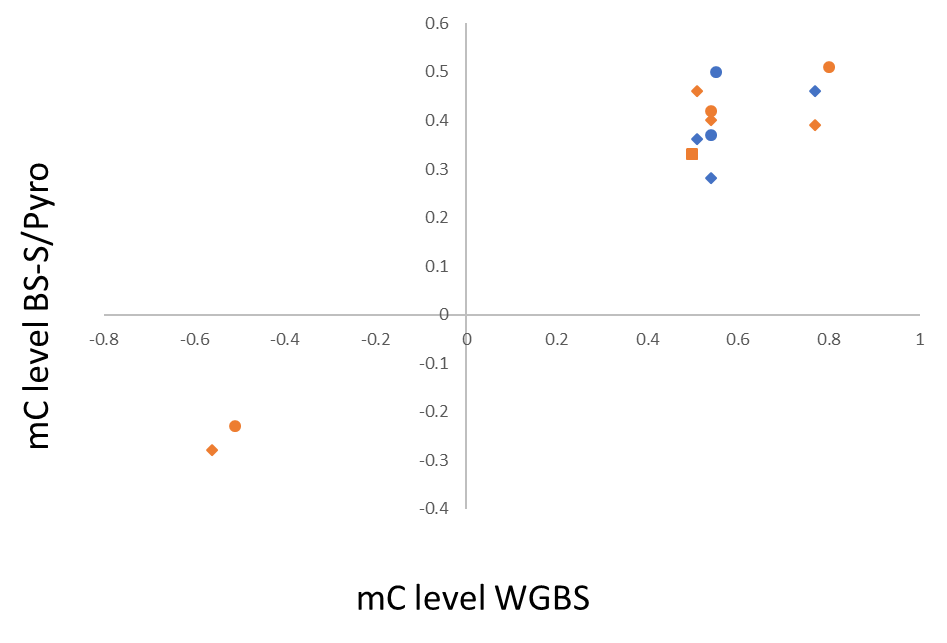
**

**Supplemental Figure S3. Reduced DNA methylation differences in monozygotic twins as compared to unrelated donors.** Example of differentially methylated sites (at 30X) along chromosome 21 in unrelated (MZ3 twin parents, unr) and MZ twins (MZ3, d3)

**
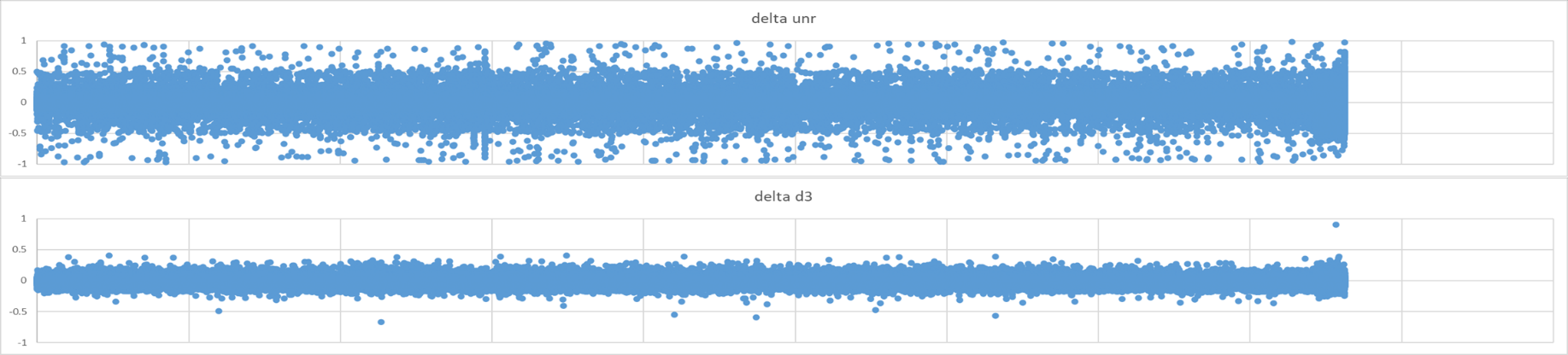
**

**Supplemental Figure S4.** Enrichment of genes matching membership terms Neural crest migration and Osteoblast differentiation. The outer pie shows the number and the percentage of genes in the background gene list (all genes with methylation data) that are associated with neural crest migration (left) or osteoblast differentiation ( right); the inner pie shows the number and the percentage of genes in the differentially methylated gene list that are associated with the membership terms. The p-value indicates whether the membership is statistically significantly enriched in the list.


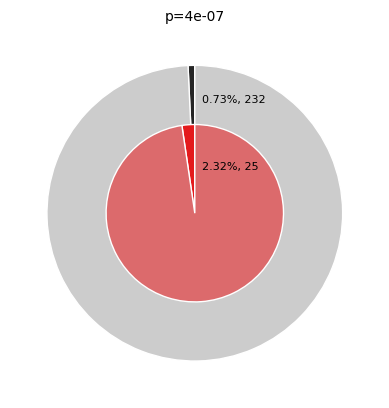

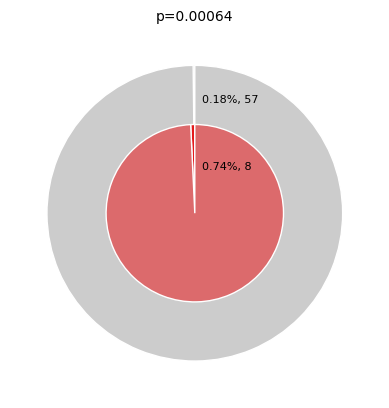


**Supplemental Figure S5. Differentially methylated genes in the Hippo pathway**


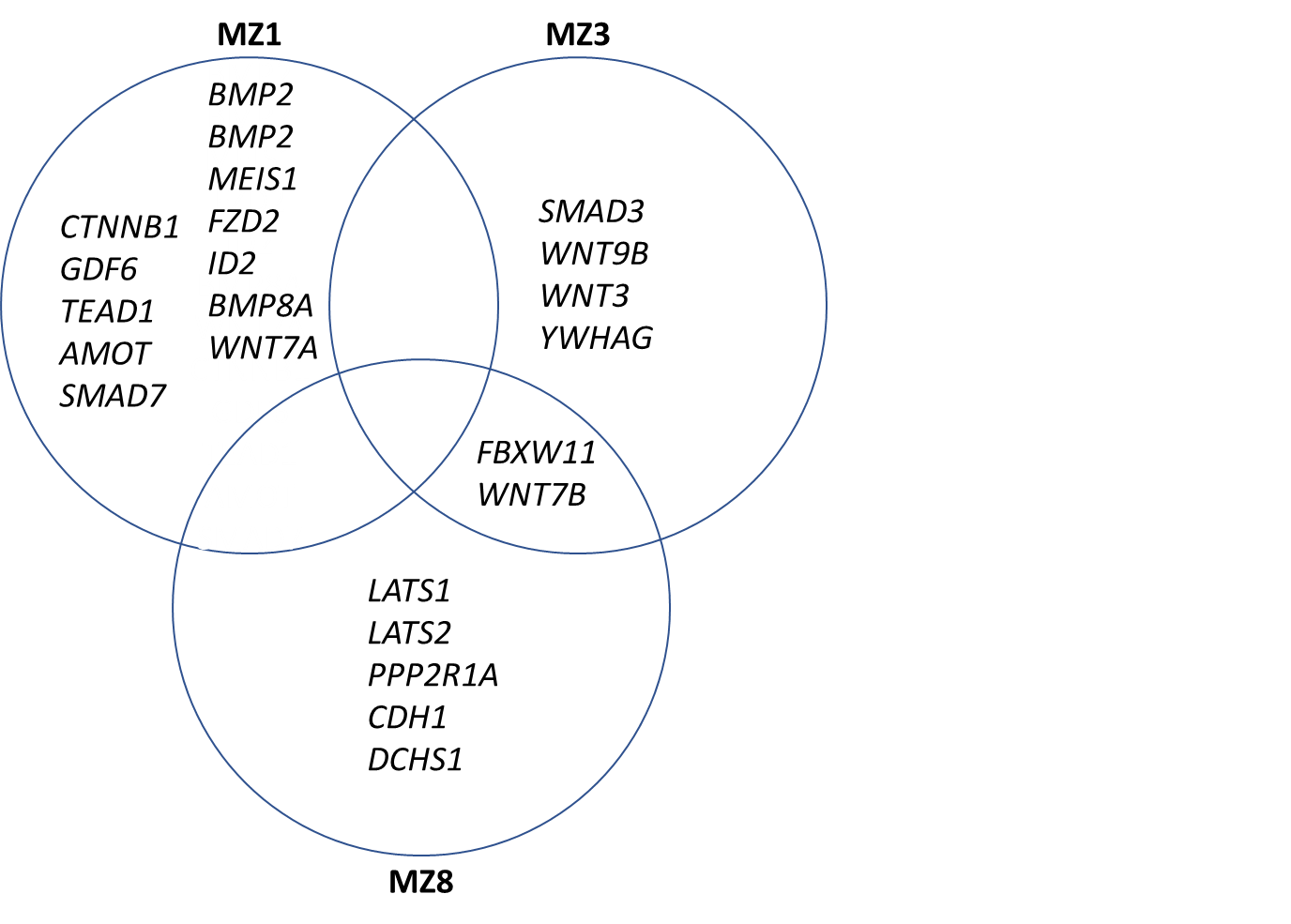


**Supplemental Table 3. Overlap between the intra-twin differentially methylated genes and the genes identified as differentially methylated by Alvizi et al (DMP in a NSCLP vs Ctrl comparison)**

| Genes in common |
| --- |
| *AGAP3* |
| *BBX* |
| *BRCA2* |
| *C9orf3* |
| *CBFA2T3* |
| *CYP11B1* |
| *DNAJC5* |
| *EPHB4* |
| *ERGIC3* |
| *FAM105B* |
| *FANCC* |
| *FAT1* |
| *FBXW11* |
| *GBX1* |
| *GPR89B* |
| *HDAC4* |
| *HIST1H2BL* |
| *KCNH1* |
| *KCNK2* |
| *KLHDC4* |
| *MAFB* |
| *MECOM* |
| *N4BP2L2* |
| *OPRK1* |
| *OSGIN2* |
| *PARP11* |
| *PDXDC1* |
| *POTEE* |
| *RREB1* |
| *SBF2* |
| *SCAND3* |
| *SEPT9* |
| *SLC7A5* |
| *TNRC6C* |
| *TRIM27* |
| *TSPAN3* |
| *TUBA3D* |
| *ZNF184* |
| *ZNF77* |
